# How to include patients’ perspectives in cognitive effects?

**DOI:** 10.1192/j.eurpsy.2023.96

**Published:** 2023-07-19

**Authors:** J. Obbels

**Affiliations:** KU Leuven, Leuven, Netherlands

## Abstract

**Abstract:**

Cognitive (dys)function after ECT is often considered as a homogeneous phenomenon across patients. However, there are important inter-individual differences, with some patients experiencing residual invalidating cognitive deficits. We present the results of a study combining both group-level and individual-level analyses of cognitive function using an extensive cognitive test battery that was assessed in 73 patients at 5 time points during their ECT care pathway. Furthermore, we explored the presence of distinct subgroups of patients with a similar cognitive trajectory over time after treatment with ECT using Latent Class Growth Analysis.

**Disclosure of Interest:**

None Declared

